# Supplementary material for: Uhrf1 regulates active transcriptional marks at bivalent domains in pluripotent stem cells through Setd1a
Source: Nat Commun. 2018 Jul 3;9:2583. doi: 10.1038/s41467-018-04818-0 (PMC6030064; doi:10.1038/s41467-018-04818-0)
Supplement: Supplementary file 1 — Supplementary Information [file 41467_2018_4818_MOESM1_ESM.pdf]

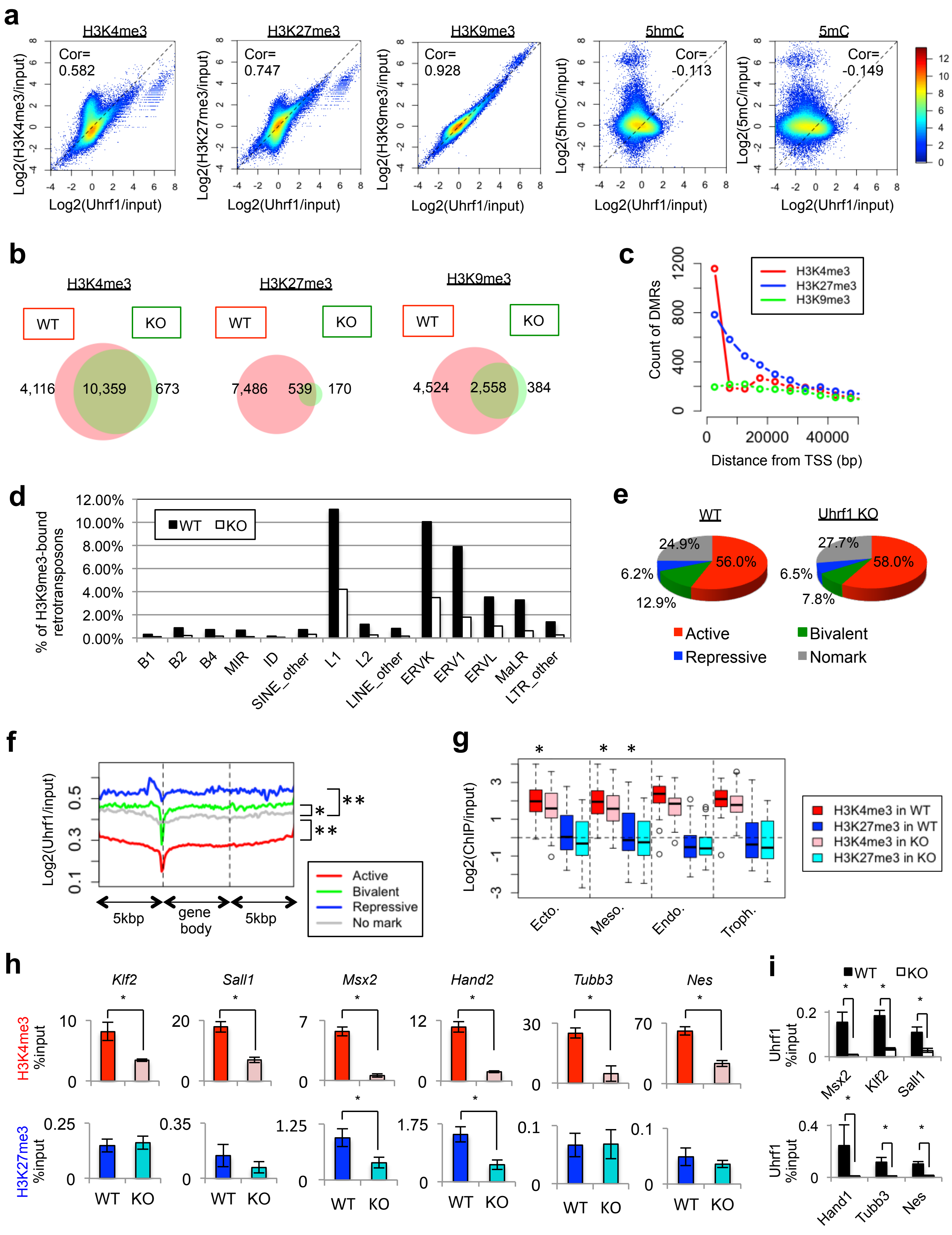

a
H3K4me3
Cor= 0.582
Log2(H3K4me3/input)
Log2(Uhrf1/input)
H3K27me3
Cor= 0.747
Log2(H3K27me3/input)
H3K9me3
Cor= 0.928
Log2(H3K9me3/input)
5hmC
Cor= -0.113
Log2(5hmC/input)
5mC
Cor= -0.149
Log2(5mC/input)
b
H3K4me3
WT
KO
4,116
10,359
673
H3K27me3
7,486
539
170
H3K9me3
4,524
2,558
384
c
Count of DMRs
Distance from TSS (bp)
H3K4me3
H3K27me3
H3K9me3
d
% of H3K9me3-bound retrotransposons
WT
KO
B1
B2
B4
MIR
ID
SINE_other
L1
L2
LINE_other
ERVK
ERV1
ERVL
MaLR
LTR_other
e
WT
Uhrf1 KO
24.9%
56.0%
6.2%
12.9%
27.7%
58.0%
6.5%
7.8%
Active
Bivalent
Repressive
Nomark
f
Log2(Uhrf1/input)
5kbp
gene body
5kbp
Active
Bivalent
Repressive
No mark
g
Log2(ChIP/input)
Ecto.
Meso.
Endo.
Troph.
H3K4me3 in WT
H3K27me3 in WT
H3K4me3 in KO
H3K27me3 in KO
h
Klf2
Sall1
Msx2
Hand2
Tubb3
Nes
H3K4me3 %input
H3K27me3 %input
WT
KO
i
WT
KO
Uhrf1 %input
Msx2
Klf2
Sall1
Hand1
Tubb3
Nes

Supplementary Figure 1. Uhrf1 is strongly associated with histone modifications

(a) Comparison of genome-wide distribution of Uhrf1 against histone modifications, 5hmC and 5mC. Log2 (ChIP/input) was calculated in 10k-bp bins throughout the mouse genome. Z-axis represents log2 (count+1). Pearson correlation coefficients are shown.

(b) Overlap of histone modification peaks between WT and Uhrf1 KO ESCs

(c) Distance of differential histone modification peaks from nearest TSSs.

(d) Percentage of H3K9me3-enriched retrotransposons in WT and Uhrf1 KO ESCs

(e) Frequency of promoter classes between WT and Uhrf1 KO ESCs.

(f) Distribution of Uhrf1 binding in active, bivalent, repressive and no mark genes. Enrichment of Uhrf1 on gene body was evaluated by two-side Student's *t*-test (* $p<0.05$ and ** $p< 0.005$).

(g) Comparison of histone modification levels in promoter regions of WT and Uhrf1 KO ESCs. Red and pink boxes represent log2 (H3K4me3 ChIP/input) in WT and Uhrf1 KO ESCs, respectively. Blue and cyan boxes represent log2 (H3K27me3 ChIP/input) in WT and Uhrf1 KO ESCs, respectively. * $p<0.05$ by one-side Student's *t*-test

(h-i) ChIP-qPCR for (h) H3K4me3, H3K27me3 and (i) Uhrf1 in pluripotent, mesodermal and neuroectodermal gene loci. * $p<0.05$ by two-side paired Student's *t*-test.

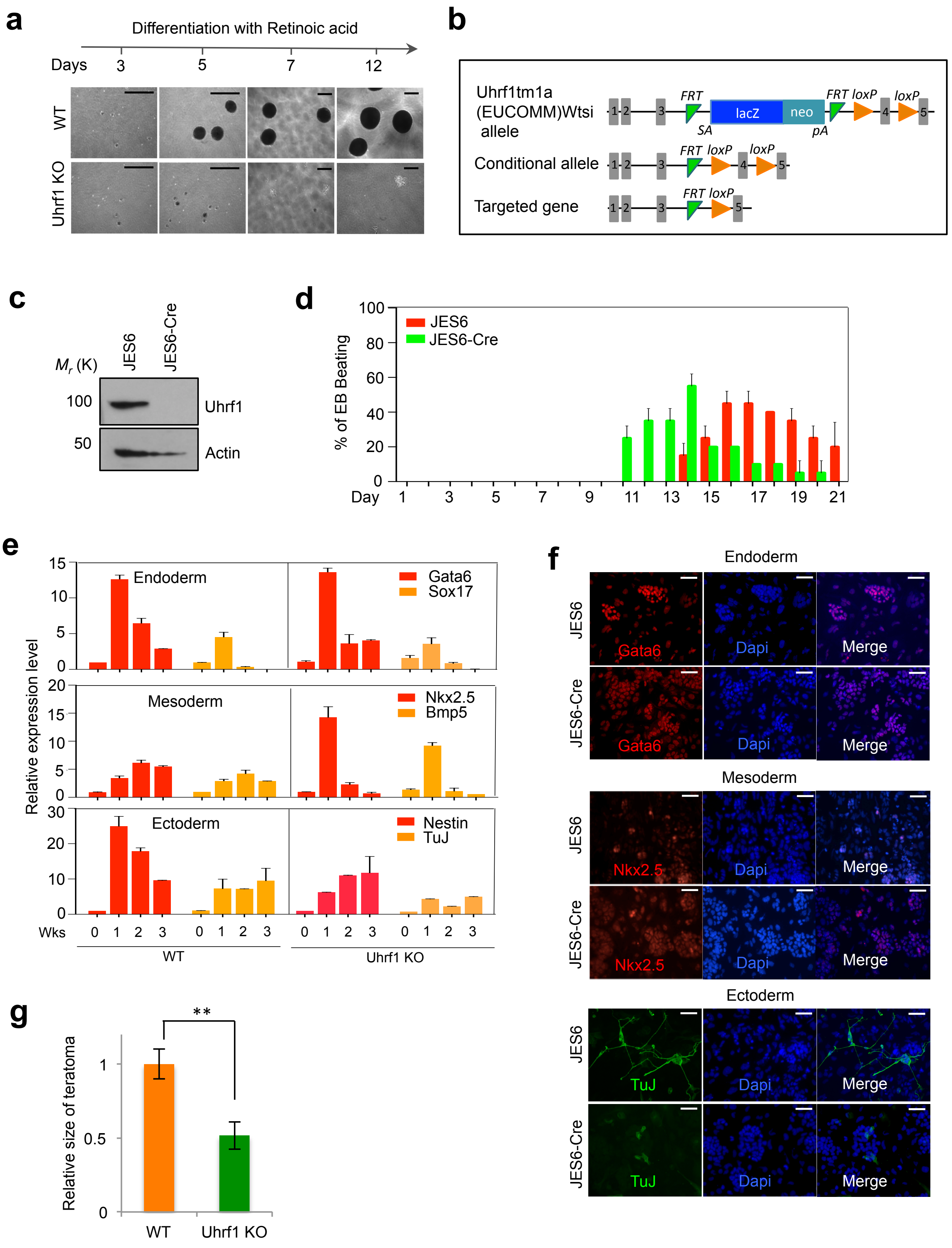
a
Differentiation with Retinoic acid
Days 3 5 7 12
WT
Uhrf1 KO
b
Uhrf1tm1a (EUCOMM)Wtsi allele
FRT
lacZ
neo
SA
pA
FRT loxP
loxP
Conditional allele
Targeted gene
c
M_r (K)
JES6
JES6-Cre
100
50
Uhrf1
Actin
d
JES6
JES6-Cre
% of EB Beating
Day
e
Endoderm
Gata6
Sox17
Mesoderm
Nkx2.5
Bmp5
Ectoderm
Nestin
TuJ
Relative expression level
Wks
WT
Uhrf1 KO
f
Endoderm
Mesoderm
Ectoderm
JES6
JES6-Cre
Gata6
Nkx2.5
TuJ
Dapi
Merge
g
**
Relative size of teratoma
WT
Uhrf1 KO

Supplementary Figure 2. Defects in differentiation potential in Uhrf1 KO mESCs.

(a) WT and KO cells were differentiated to EBs in the presence of neural lineage specific differentiation medium supplemented with retinoic acid. The scale bar represents 4 mm.

(b) The structure of the Uhrf1 tm1a(EUCOMM) Wtsi allele. Excising the FRT with Flippase created a conditional allele. Expression of Cre recombinase excised exon 4 of Uhrf1.

(c) Western blotting analysis of Uhrf1 in JES6-Cre mESC line.

(d) JES6 and JES6-Cre mESCs were differentiated to EBs, and analyzed daily under the microscope for the presence of a rhythmic beating until day 21. Data represent the mean ± SD of three independent experiments.

(e) qPCR analysis of lineage specific and pluripotency markers upon *in vitro* differentiation. Three independent experiments were carried out and values were normalized to β-actin. The error bars represent the standard deviation.

(f) Representative Immunostaining of differentiated JES6 and JES6-Cre ESC lines. One week post differentiation, EBs was dissociated and plated onto gelatin-coated plates. Immunostaining with lineage specific markers was performed 2 days after plating. The scale bar represents 4 mm.

(g) Quantification of teratoma diameter generated from WT or Uhrf1 KO ESCs. Data represent the mean ± SD of six independent experiments. ** $p<0.01$ by unpaired, two-tailed Student's *t*-test analysis.

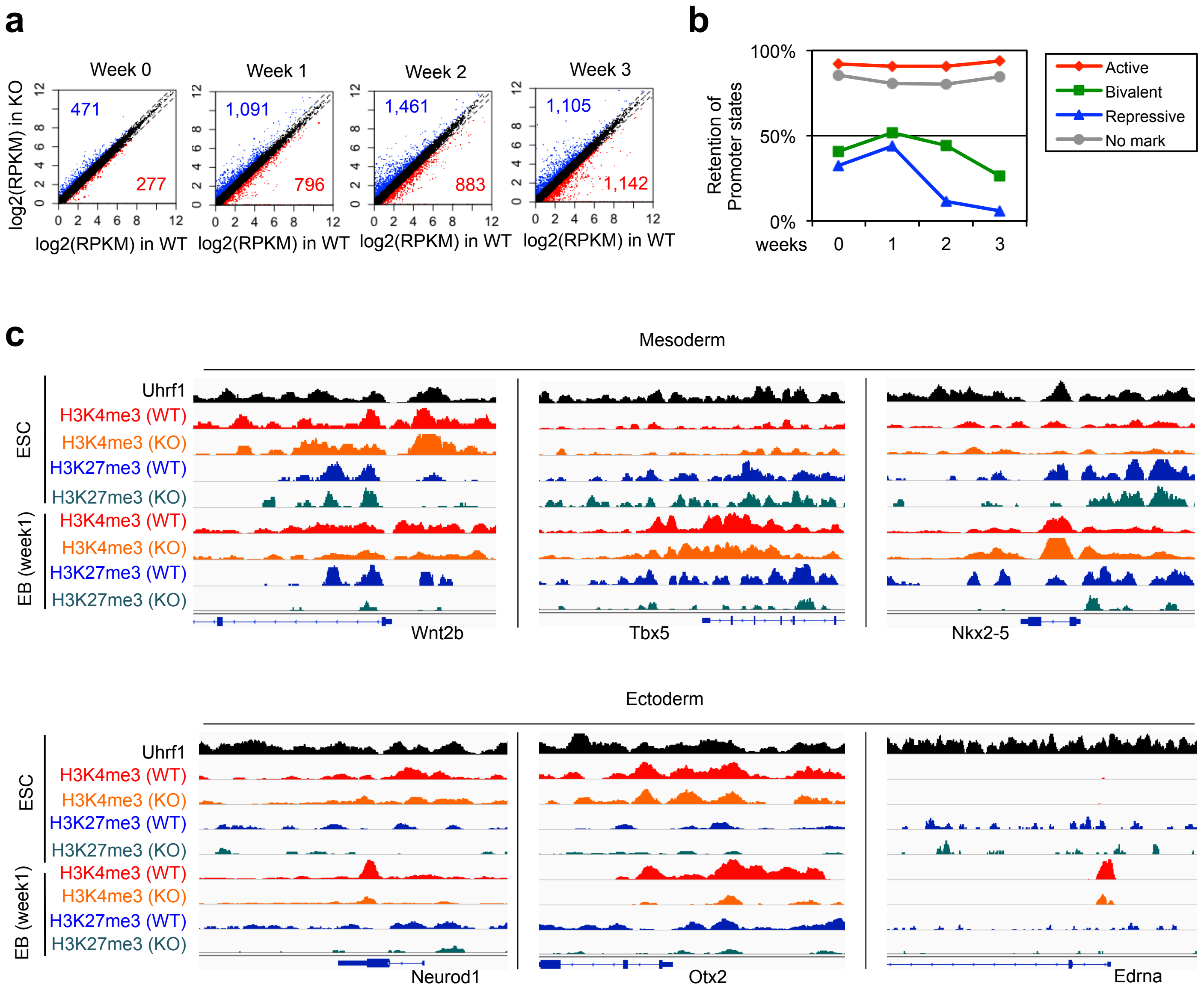

Supplementary Figure 3. Disruption of histone modifications in Uhrf1 KO mESCs.

(a) Comparison of gene expression profiles between WT and Uhrf1 KO ESCs at each time point. Red and blue dots represent differentially expressed genes (>1.5 fold) in WT and KO, respectively. The count represents the number of differentially expressed genes.

(b) Percentage of promoter states retained in Uhrf1 KO at each time point.

(c) Histone modification landscape of representative genes related to mesodermal, endodermal, and neuroectodermal development in ESCs and week 1 EBs.

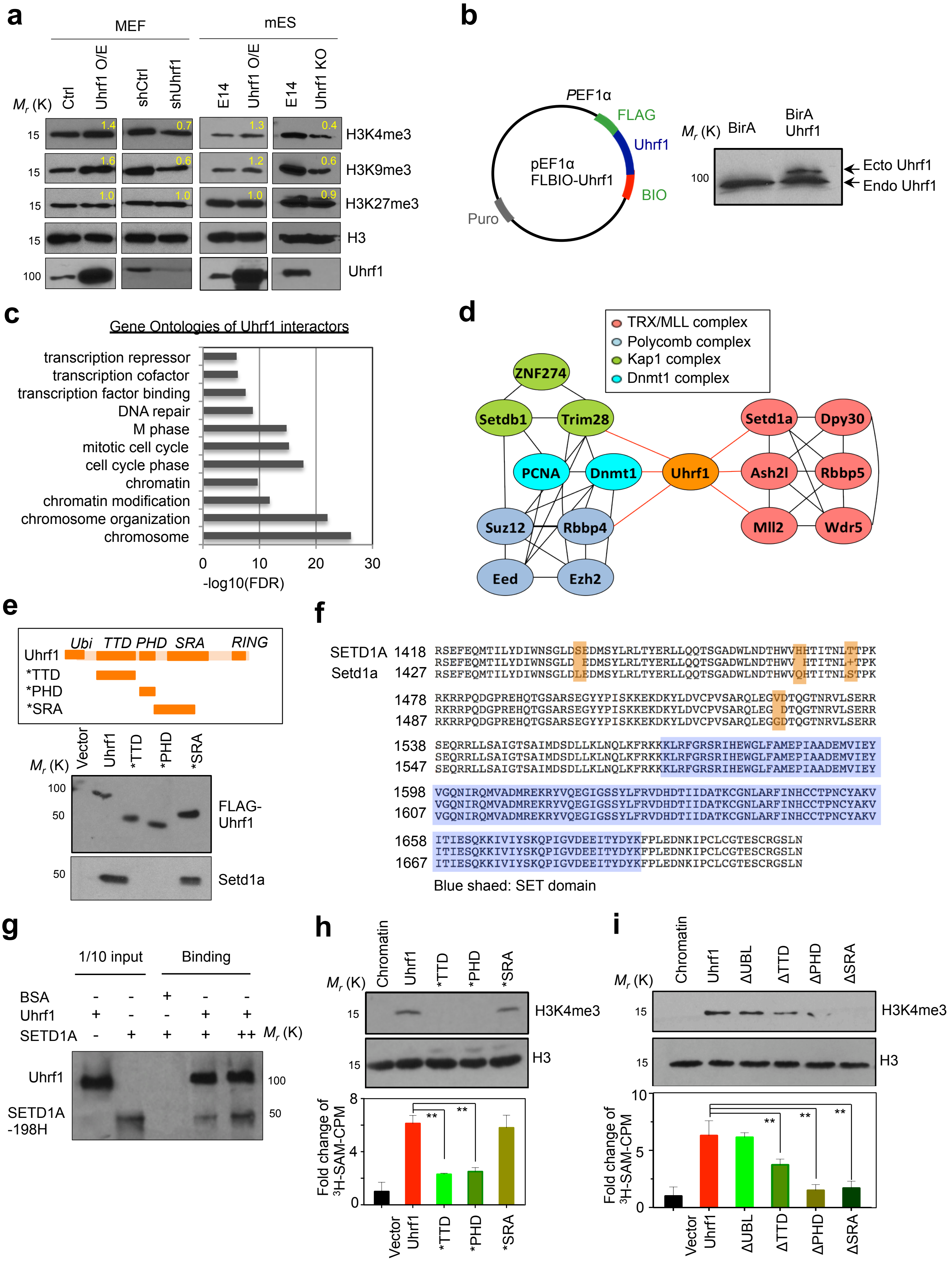
a
MEF
mES
Mr (K)
Ctrl
Uhrf1 O/E
shCtrl
shUhrf1
E14
Uhrf1 O/E
E14
Uhrf1 KO
H3K4me3
H3K9me3
H3K27me3
H3
Uhrf1
b
PEF1α
FLAG
Uhrf1
BIO
Puro
pEF1α FLBIO-Uhrf1
BirA
BirA Uhrf1
Ecto Uhrf1
Endo Uhrf1
c
Gene Ontologies of Uhrf1 interactors
transcription repressor
transcription cofactor
transcription factor binding
DNA repair
M phase
mitotic cell cycle
cell cycle phase
chromatin
chromatin modification
chromosome organization
chromosome
-log10(FDR)
d
TRX/MLL complex
Polycomb complex
Kap1 complex
Dnmt1 complex
ZNF274
Setdb1
Trim28
PCNA
Dnmt1
Uhrf1
Suz12
Rbbp4
Eed
Ezh2
Setd1a
Dpy30
Ash2l
Rbbp5
Mll2
Wdr5
e
Ubi TTD PHD SRA RING
Uhrf1
*TTD
*PHD
*SRA
Vector
FLAG-Uhrf1
Setd1a
f
SETD1A 1418 RSEFEQMTILYDIWNSGLDSEDMSYLRLTYERLLQQTSGADWLNDTHWVHHTITNLTTPK
Setd1a 1427 RSEFEQMTILYDIWNSGLDLEDMSYLRLTYERLLQQTSGADWLNDTHWVQHTITNLSTPK
1478 RKRRPQDGPREHQTGSARSEGYYPISKKEKDKYLDVCPVSARQLEGVDTQGTNRVLSERR
1487 RKRRPQDGPREHQTGSARSEGYYPISKKEKDKYLDVCPVSARQLEGGDTQGTNRVLSERR
1538 SEQRRLLSAIGTSAIMDSDLLKLNQLKFRKKKLRFGRSRIHEWGLFAMEPIAADEMVIEY
1547 SEQRRLLSAIGTSAIMDSDLLKLNQLKFRKKKLRFGRSRIHEWGLFAMEPIAADEMVIEY
1598 VGQNIRQMVADMREKRYVQEGIGSSYLFRVDHDTIIDATKCGNLARFINHCCTPNCYAKV
1607 VGQNIRQMVADMREKRYVQEGIGSSYLFRVDHDTIIDATKCGNLARFINHCCTPNCYAKV
1658 ITIESQKKIVIYSKQPIGVDEEITYDYKFPLEDNKIPCLCGTESCRGSLN
1667 ITIESQKKIVIYSKQPIGVDEEITYDYKFPLEDNKIPCLCGTESCRGSLN
Blue shaed: SET domain
g
1/10 input
Binding
BSA
Uhrf1
SETD1A
SETD1A -198H
h
Chromatin
Uhrf1
*TTD
*PHD
*SRA
H3K4me3
H3
Fold change of ³H-SAM-CPM
Vector
i
Chromatin
Uhrf1
ΔUBL
ΔTTD
ΔPHD
ΔSRA
H3K4me3
H3
Fold change of ³H-SAM-CPM
Vector

Supplementary Figure 4. The SRA of Uhrf1 mediates the direct interaction with Setd1a.

(a) Western blotting analysis of H3K4me3, H3K27me3 and H3K9me3. Overexpression and knock down of Uhrf1 in MEFs and mESCs were analyzed (Experimental replicates, n=3). Quantification of band intensity was analyzed using Image J software, and relative expression of Uhrf1 compared with control O/E, control shRNA or WT ESC is shown with mean +/- S.D. * $p<0.05$ by unpaired, two-tailed Student's *t*-test analysis.

(b) Establishment of mESC lines stably expressing Uhrf1 with FLAG tag. Full length Uhrf1 was subcloned into a pEF1alphaFLAGBio (FLBIO)-puro vector using SmaI and EcoRV. Western blot analysis using Uhrf1 antibody confirmed the expression of sub-endogenous level of ectopic Uhrf1.

(c) Over represented GO term of Uhrf1 interaction partners

(d) Network analysis of Uhrf1 histone modification complexes. Red edges are interactions detected in this study. Black edges are interactions in BioGRID database (version 3.4.125).

(e) Each domain of Uhrf1 (*TTD, *PHD and *SRA) was expressed in HEK293T cells and used for co-immunoprecipitation with Setd1a. The SRA domain is sufficient to interact with Setd1a.

(f) Alignment of human SETD1A-198H (amino acid 1418 – end) and murine Setd1a (amino acid 1427 – end). The blue shaded amino acids represent SET domain, which are identical in both mouse and human. Orange box represents the different amino acid.

(g) Uhrf1 directly interacts with SETD1A. Purified recombinant Uhrf1 and recombinant partial SETD1A protein SETD1A-198H (amino acid 1418 – end) that has n-SET and SET domain (f), were used in an *in vitro* binding assay. For input, 1/10 of proteins used for binding assay were loaded. For binding, + represents adding of 0.15 mg, and ++ represents 0.3 mg. After pulling down recombinant Uhrf1 with an Uhrf1 antibody, a GST antibody was used for Western analysis to detect Uhrf1 and SETD1A-198H.

(h) *In vitro* HMT assay performed with protein complexes, which had been immunoprecipitated with each of the Uhrf1 domain shown in (c). The complex from SRA domain of Uhrf1 was sufficient for H3K4me3 modification of chromatin substrate.

(i) The TTD and PHD domain in addition to the SRA domain of Uhrf1 are essential for full methylation activity towards chromatin as a substrate.

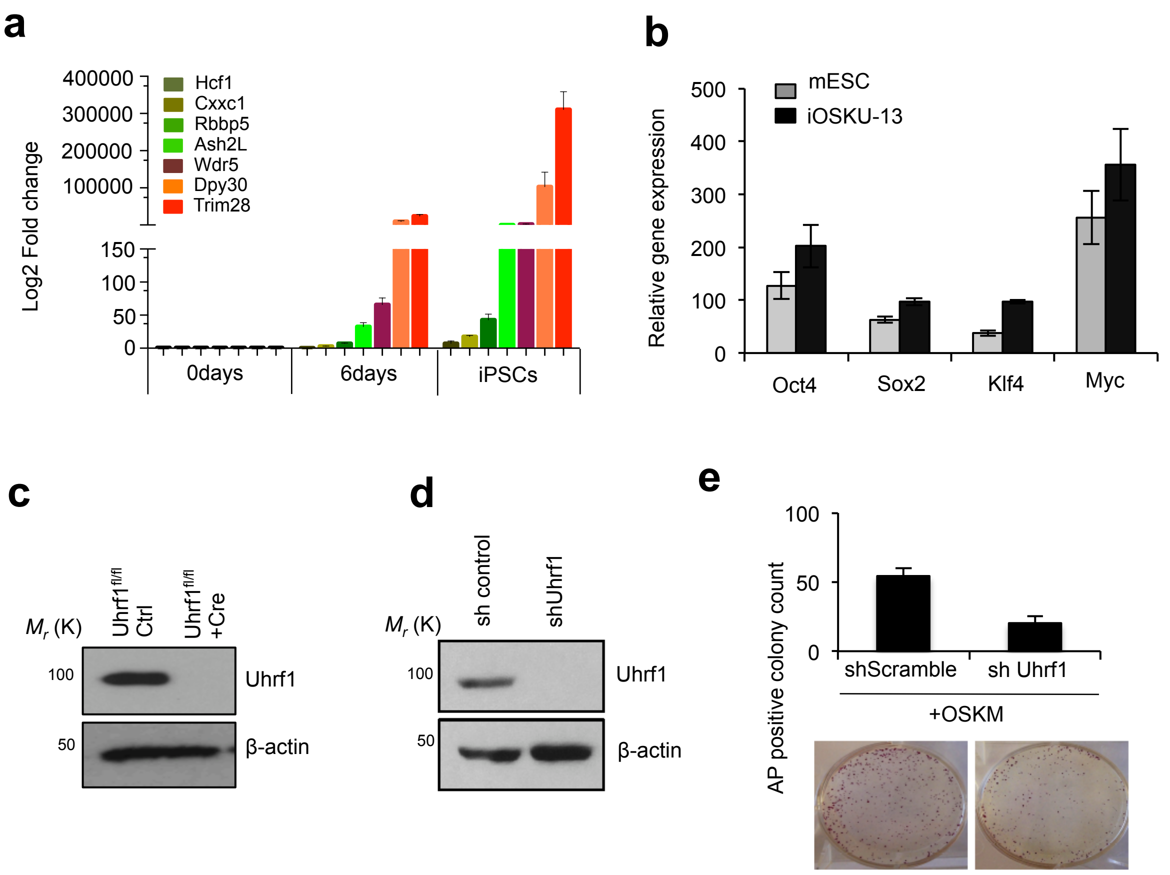

Supplementary Figure 5. Uhrf1 is essential for reprogramming.

(a) Expression of Setd1a/COMPASS complex proteins during reprogramming. Data are expressed relative to Day 0 MEF values. The data is from *n* = 3 experimental replicates.

(b) Expression of endogenous pluripotent genes (*Oct4*, *Sox2*, *Klf4*, and *Myc*) in iPSC line derived from MEFs by expression of Oct4, Sox2, Klf4 and Uhrf1 (iOSKU-13) compared with those in J1 mESC line.

(c) Deletion of Uhrf1 in Uhrf1$^{fl/fl}$-MEFs by expression of Cre recombinase validated by Western analysis.

(d) Depletion of Uhrf1 using shRNA in MEFs was confirmed using Western analysis.

(e) Quantification of Alkaline Phosphatase positive colonies on reprogramming day 13 after expressing four reprogramming factors (Oct4, Sox2, Klf4 and Myc, OSKM) in MEFs infected with lentivirus expressing scramble or Uhrf1 shRNAs.

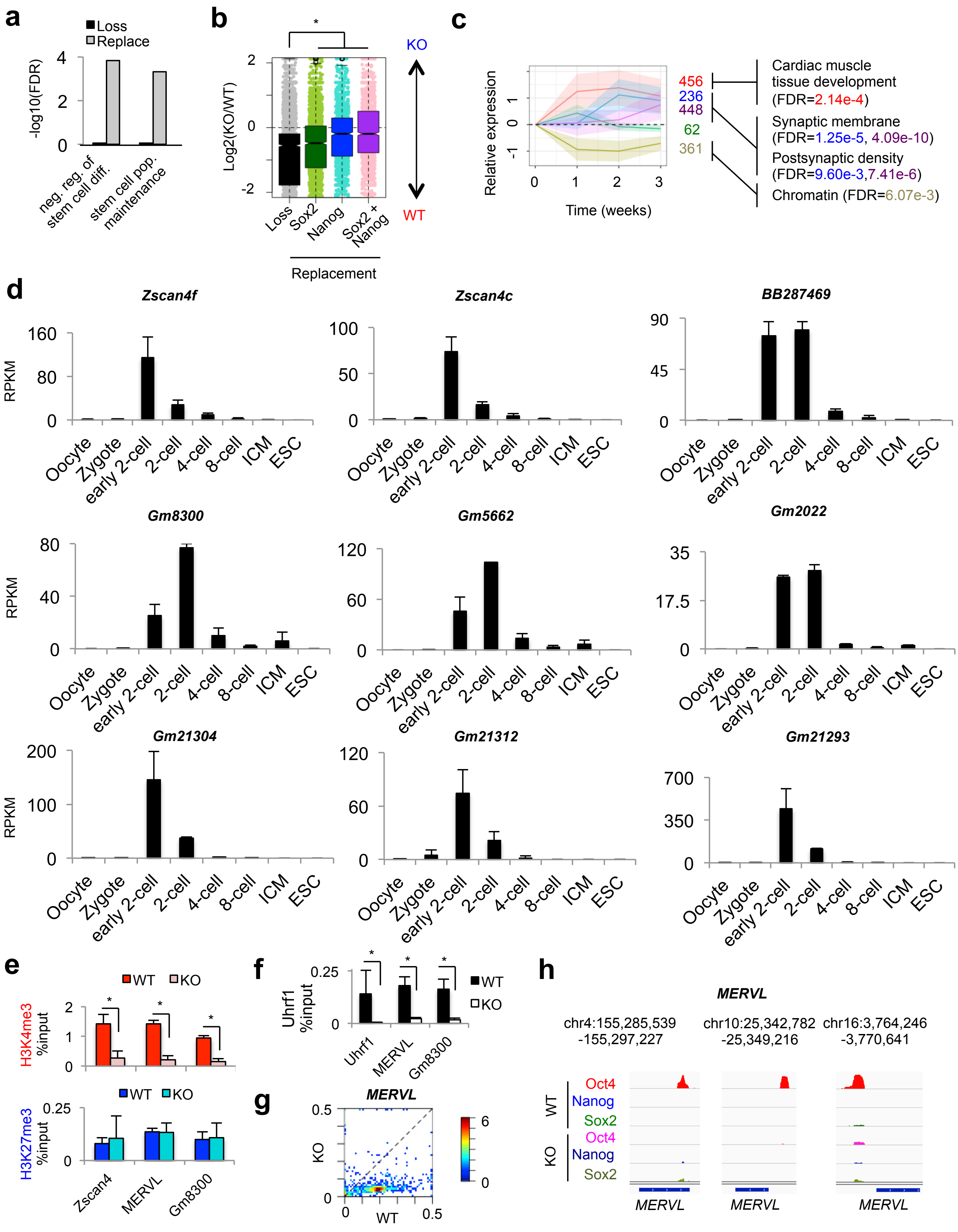
a
Loss
Replace
-log10(FDR)
neg. reg. of stem cell diff.
stem cell pop. maintenance
b
Log2(KO/WT)
KO
WT
Loss
Sox2
Nanog
Sox2 + Nanog
Replacement
c
Relative expression
Time (weeks)
456
236
448
62
361
Cardiac muscle tissue development (FDR=2.14e-4)
Synaptic membrane (FDR=1.25e-5, 4.09e-10)
Postsynaptic density (FDR=9.60e-3,7.41e-6)
Chromatin (FDR=6.07e-3)
d
Zscan4f
Zscan4c
BB287469
Gm8300
Gm5662
Gm2022
Gm21304
Gm21312
Gm21293
RPKM
Oocyte
Zygote
early 2-cell
2-cell
4-cell
8-cell
ICM
ESC
e
WT
KO
H3K4me3 %input
H3K27me3 %input
Zscan4
MERVL
Gm8300
f
Uhrf1 %input
Uhrf1
MERVL
Gm8300
g
MERVL
KO
WT
h
MERVL
chr4:155,285,539 -155,297,227
chr10:25,342,782 -25,349,216
chr16:3,764,246 -3,770,641
WT
KO
Oct4
Nanog
Sox2
MERVL

Supplementary Figure 6. Uhrf1 regulates 2C-related genes.

(a) Functions of genes that show a loss or replacement in Oct4 binding upon Uhrf1 KO are related with stem cell maintenance. –log10 (FDR) for GO terms, GO:2000737 (negative regulation of stem cell differentiation) and GO:0019827 (stem cell population maintenance), is shown.

(b) Comparison of chromatin accessibility between WT and KO in loss or replacement of Oct4 binding sites. * $p<0.05$ by two-side Student's *t*-test.

(c) Gene expression patterns of target genes where loss of Oct4 binding has occurred. Target genes were clustered by Pearson correlation coefficient. The number of genes and representative GO terms in each cluster were also shown in the right panel.

(d) 2C stage-specific gene expression of top down-regulated genes in Uhrf1 KO ESCs.

(e-f) ChIP-qPCR for (e) H3K4me3, and H3K27me3, or (f) Uhrf1 on 2C-gene loci. * $p<0.05$ by two-side Student's *t*-test .

(g) Comparison of MERVL retrotransposon expression between WT and Uhrf1 KO ESCs.

(h) Binding landscape of core pluripotent factors around *MERVLs*.

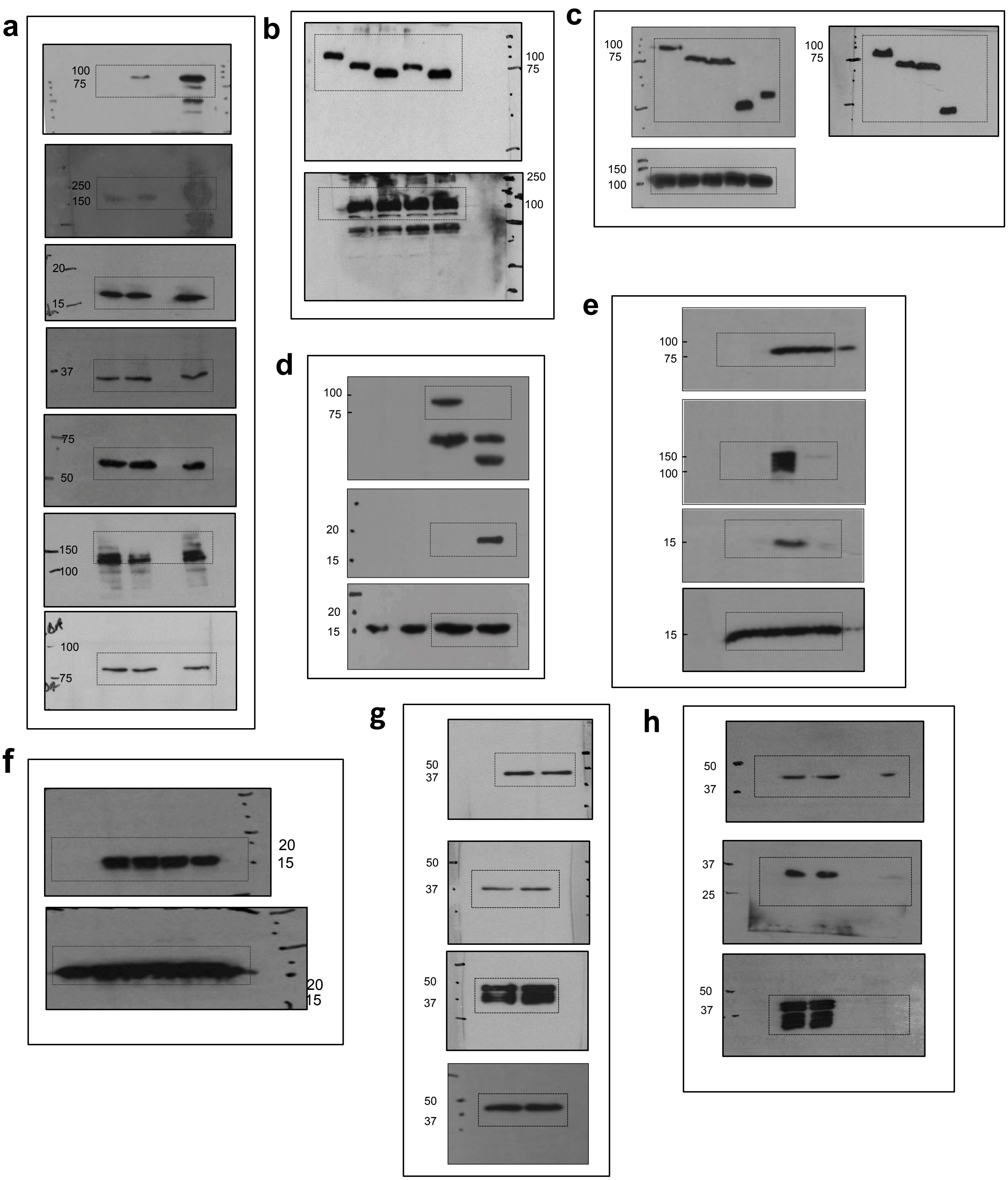

Supplementary Figure 7. Uncropped scans of immunoblots related to main figures.

The corresponding figure number and the molecular weight in kDa are indicated. Dot-lined boxes indicate the cropped areas presented in the figures. **(a)** for Fig. 3a, **(b)** for Fig. 3b, **(c)** for Fig. 3c, **(d)** for Fig. 3d, **(e)** for Fig. 3e, **(f)** for Fig. 3f, **(g)** for Fig. 5a, **(h)** for Fig. 5b.

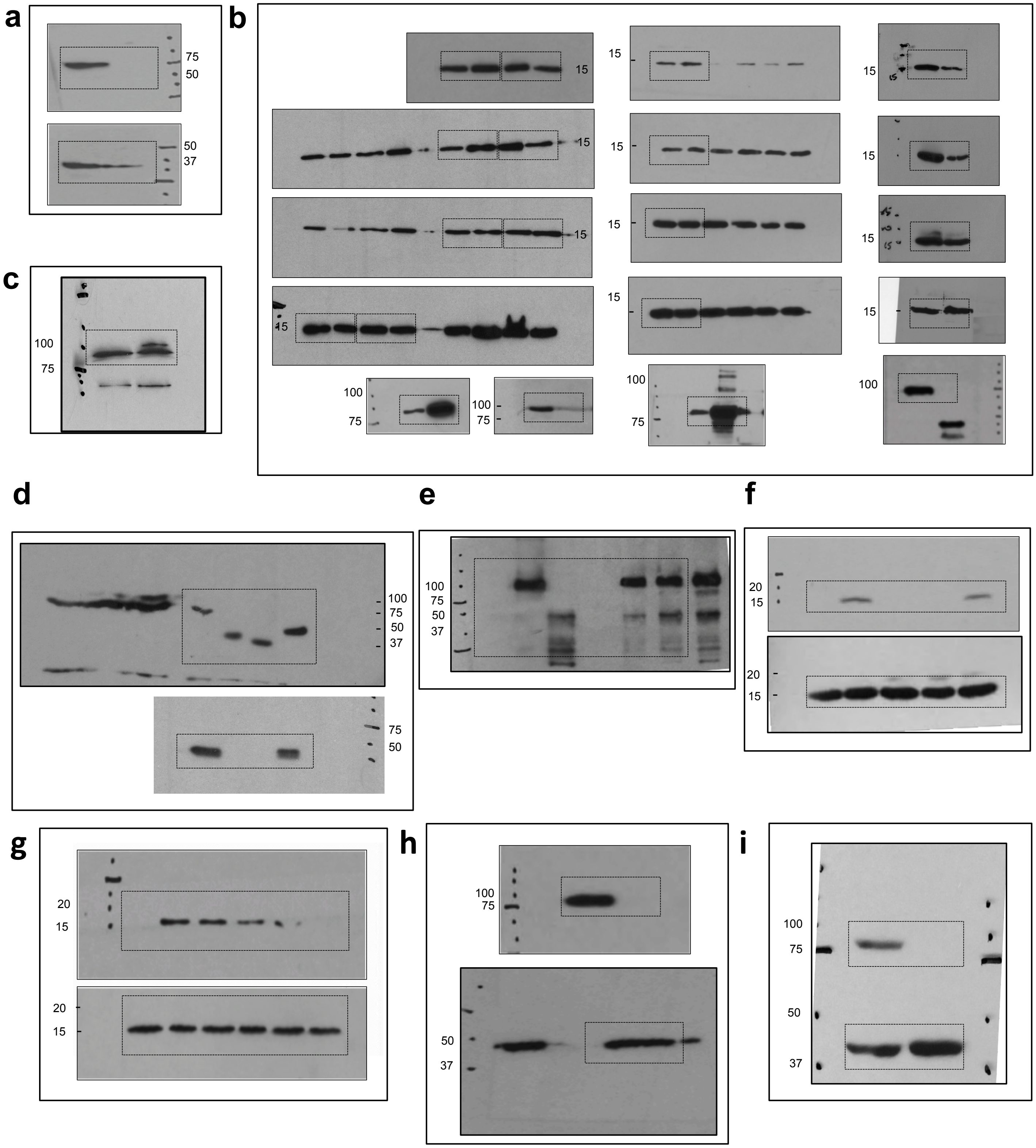

Supplementary Figure 8. Uncropped scans of immunoblots related to suppl. figures.

The corresponding figure number and the molecular weight in kDa are indicated. Dot-lined boxes indicate the cropped areas presented in the figures. **(a)** for Supp. Fig. 2c, **(b)** for Supp. Fig. 4a, **(c)** for Supp. Fig. 4b, **(d)** for Supp. Fig. 4c, **(e)** for Supp. Fig. 4e, **(f)** for Supp. Fig. 4f, **(g)** for Supp. Fig. 4g, **(h)** for Supp. Fig. 5c, **(i)** for Supp. Fig. 5d.
