# Supplementary material for: Uhrf1 regulates active transcriptional marks at bivalent domains in pluripotent stem cells through Setd1a
Source: Nat Commun. 2018 Jul 3;9:2583. doi: 10.1038/s41467-018-04818-0 (PMC6030064; doi:10.1038/s41467-018-04818-0)
Supplement: Supplementary file 3 — Description of Additional Supplementary Files [file 41467_2018_4818_MOESM3_ESM.pdf]

Description of Additional Supplementary Files

File Name: Supplementary Data 1
Description: Uhrf1-interacting proteins detected by LC-MS/MS.

File Name: Supplementary Data 2
Description: Primers used for RT-qPCR and ChIP-qPCR.

File Name: Supplementary Data 3
Description: Antibodies used for this study.

File Name: Supplementary Data 4
Description: Summary of high-throughput sequencing.
